# Potential Region-Specific Neuroprotective Effects of Kynurenine Administration in Healthy Rodents Using High-Resolution Mass Spectrometry

**DOI:** 10.1021/acschemneuro.4c00586

**Published:** 2025-09-15

**Authors:** Sandy Abujrais, Anne Simeit, Mara Link, Fleur Kalberg, Leandrie Pienaar, Radhini Veerappan, Aletta ME Millen, Sooraj Baijnath, Jonas Bergquist

**Affiliations:** a Analytical Chemistry and Neurochemistry, Department of Chemistry−BMC, 8097 Uppsala University, Uppsala 75124, Sweden; b The ME/CFS Collaborative Research Centre, 8097 Uppsala University, Uppsala 75124, Sweden; c Wits Integrated Molecular Physiology Research Initiative, Wits Health Consortium (PTY) Ltd., School of Physiology, Faculty of Health Sciences, 37707 University of The Witwatersrand, Johannesburg 2050, South Africa; d School of Physiology, Faculty of Health Sciences, 37707 University of The Witwatersrand, Private Bag 3, Wits, Johannesburg 2050, South Africa

**Keywords:** tryptophan metabolism, kynurenine, kynurenine administration, kynurenic acid, neuroprotection, inflammation, neurological disorders

## Abstract

The tryptophan (TRP) metabolic pathway produces kynurenine (KYN) and serotonin (5-HT). These are important molecules in the central nervous system, as KYN plays a crucial role in neuroprotection, while 5-HT impacts mood and sleep patterns. The production of KYN is increased in response to inflammatory cytokines and cortisol release, which activates indoleamine 2,3-dioxygenase (IDO) and tryptophan 2,3-dioxygenase (TDO), respectively. These enzymes are responsible for converting TRP and KYN into neuroactive molecules including kynurenic acid (KA), quinolinic acid (QA), and 3-hydroxykynurenine (3HK). These metabolites play an important role in neuroprotection and have been linked to the development of several neurological disorders. Therefore, the aim of this study was to investigate the effect of exogenous KYN administration on the activity of the KYN pathway by measuring the brain tissue concentration of these metabolites and the mRNA expression of inflammatory markers, neurotrophic factors, IDO, and TDO. In the acute study, Sprague–Dawley rats (*n* = 25) received 100 mg/kg kynurenine (0.2 mL, ip) and were terminated at *t* = 0.5, 1, 2, 3, and 5 h post-KYN administration (*n* = 5/time point) while in the control group (*n* = 5) received saline (0.2 mL, ip) and were terminated at *t* = 1 h. In the chronic study, both KYN and control animals (*n* = 6 per group) received the same dose as the acute study for 14 days, once daily. Following the treatment period, animals were terminated by decapitation, and trunk blood was collected and separated into plasma, while the brain was surgically removed and dissected into the hippocampus, hypothalamus, midbrain, prefrontal cortex, striatum, cortex, and cerebellum. KYN metabolites were measured by liquid chromatography coupled to high-resolution mass spectrometry, while the mRNA expression of *IDO*, *TDO*, brain-derived neurotrophic factor (*BDNF*), cAMP response element-binding protein (*CREB*), and interleukin-6 (*IL-6*) was measured using RT-PCR. KYN and its metabolites were quantified at basal levels in plasma and seven brain regions to assess their distribution in the peripheral and central nervous system. The KA/3HK ratio increased in multiple brain regions, and the plasma KA/QA ratio increased significantly after acute and chronic KYN administration, suggesting peripheral neuroprotection. Reduced plasma and cerebellar KA/3HK ratios suggest region-specific neurotoxicity, whereas the hippocampus accumulates the most KYN and its metabolite KA, suggesting the potential neuroprotective effect of KYN administration in the hippocampus.

## Graphical abstract

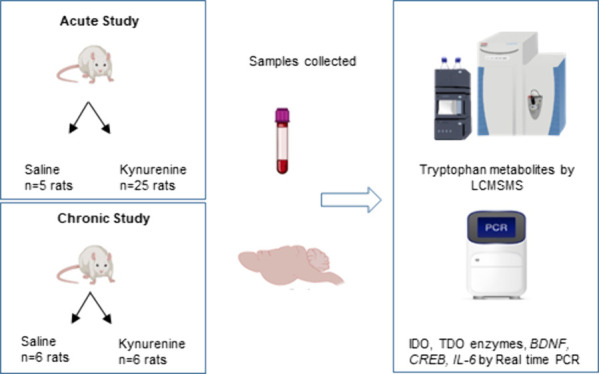

## Introduction

Kynurenine (KYN), together with serotonin (5-HT), is the major product of the tryptophan (TRP) metabolic pathway. This is an important biosynthetic pathway responsible for the production of many essential biomolecules that regulate several important physiological processes, especially in the nervous system. While 5-HT influences mood, KYN is an important regulator of immune and neurological function.^1^ KYN production is stimulated by inflammatory cytokines such as interleukin (IL)-6, interferon (IFN)-γ, tumor necrosis factor (TNF)-α, and stress-induced cortisol release. These cytokines in turn activate the enzyme indoleamine 2,3-dioxygenase (IDO), while stress hormones such as cortisol can activate tryptophan 2,3-dioxygenase (TDO). IDO and TDO activation stimulates TRP metabolism leading to the production of KYN, which is then further metabolized into several neuroactive compounds such as quinolinic acid (QA), kynurenic acid (KA), or 3-hydroxykynurenine (3HK)^2,3^ (Figure S1). The end product of the KYN pathway is nicotinamide adenine dinucleotide (NAD+), a key enzymatic cofactor essential for cellular energy production,^4^ highlighting the importance of the KYN pathway in maintaining normal physiological function.

Being an important regulator of central nervous system (CNS) activity, dysregulation of the KYN pathway has been linked to the development of several neurological disorders, including anxiety, schizophrenia, and bipolar mood disorder.^5^ In addition, studies have also shown that changes in specific metabolite ratios are implicated in different disorders, such anxiety and stress-related disorders being strongly linked to imbalanced KA and QA ratios.^6^ Furthermore, increased KYN and QA production associated with the chronic activation of IDO has been linked to fatigue and depression associated with multiple sclerosis, via the alteration of the brain’s excitotoxic equilibrium and normal serotonin and melatonin production.^7^ A similar phenomenon has been observed in individuals with autism spectrum disorder where IDO activation is associated with increased KYN concentrations in the brain.^8^ Despite these disorders being associated with elevated levels of the same metabolite, they present with a different clinical picture; this can be attributed to region-specific pathological processes. For instance, disorders characterized by deficits in higher executive function show metabolomic alterations in the prefrontal cortex (PFC) and hippocampus (HIP), which includes depressive disorders and Alzheimer’s disease.^9,10^ While movement disorders, such as Parkinson’s disease, show neurodegeneration in the striatum (STR) and midbrain (MID), evidence suggests that an imbalance between specific kynurenine metabolites may be an underlying mechanism in the development of neurological disorders.^9^

During chronic inflammatory responses, which in many cases drive neurodegeneration, the ratio of KA/QA is biased toward the production of neurotoxic QA, via the microglial activation of kynurenine monooxygenase (KMO).^11^ KA is produced in astrocytes and generally regarded as a neuroprotective factor, acting as the only endogenous neuronal *N*-methyl-d-aspartate (NMDA) receptor antagonist at the glycine binding site.^12^ On the other hand, QA acts as a weak agonist, potentially inducing hyperactivity of these receptors, by increasing the risk of glutamate excitotoxicity, thereby accelerating oxidative damage and subsequent neurodegeneration.^12,13^ Apart from QA, other microglial kynurenine-related metabolites, such as 3HK and 3-hydroxyanthranilic acid (3HAA), have been shown to display neurotoxic effects due to their ability to generate free radicals and act as agonists of the NMDA receptor.^14^ In addition, inflammation associated with increased KYN pathway activity has been associated with the decreased brain-derived neurotrophic factor (*BDNF*) and cAMP response binding element (*CREB*) expression. These are important neurotrophic transcription factors that regulate neuroprotection, neuronal health, and synaptic plasticity. Taken together, this highlights the dual neuroprotective and neurodegenerative role of the KYN pathway in the brain, emphasizing the need to study KYN metabolism in different brain compartments. Given these findings, targeting the KYN pathway via exogenous KYN administration may offer therapeutic benefits such as enhancing KA production to counteract neurotoxicity or inhibiting the production of neurotoxic kynurenines like QA.

In recent years, several studies have investigated various aspects of TRP metabolism, in particular the KYN pathway.^5,15–19^ The majority of these studies focus on the analysis of serum or plasma or the whole brain and analyze almost exclusively TRP and its direct metabolites. In addition, many acute KYN studies are behavioral and cognitive studies and do not report any or only very limited analyte concentrations.^20,21^ Considering that many neurological diseases have been linked to dysregulated KYN metabolism, there is an obvious gap of knowledge for the analysis of KYN and its metabolites within specific brain regions. For example, a study by Pocivavsek et al. has analyzed acute KYN administration in specific brain regions, such as the HIP and cortex (COR).^21^ However, there is still very limited literature investigating the effects of KYN administration in other brain regions such as the striatum, hypothalamus, midbrain, prefrontal cortex, and cerebellum, which are implicated in the development of neurological diseases.

Therefore, the aim of this study is to increase our understanding of kynurenine metabolism by investigating the possible neuroprotective effects of acute and chronic KYN administration in a healthy rat model. Investigating the effect of exogenous KYN administration on the activity of the KYN pathway in a healthy system is essential for establishing a baseline from which potential KYN-based pharmaceutical treatments could be developed and tested in diseased animal models. Our findings present an overview of the pharmacokinetics of several metabolites and enzyme activity linked to the KYN pathway, which may have implications for the treatment of disorders associated with dysfunctional kynurenine metabolism.

## Results and Discussion

TRP metabolism and the KYN pathway are important regulators of physiological function via the biosynthesis of several key biomolecules. In this study, we evaluated changes in the metabolites formed by these pathways following the intraperitoneal injection (ip) of KYN. Using a targeted multiplexed method, we were able to identify changes in these metabolites in plasma and different brain regions, following acute and chronic administration. In order to understand the basal levels of both the TRP- and KYN-associated metabolites in the various brain regions, baseline concentrations were determined in the control animals who received saline (Figure 1). High-resolution mass spectrometry analysis showed the preferential regional distribution of several metabolites, demonstrating their ability to cross the blood–brain barrier, even when KYN is administered systemically.

**Figure 1:**
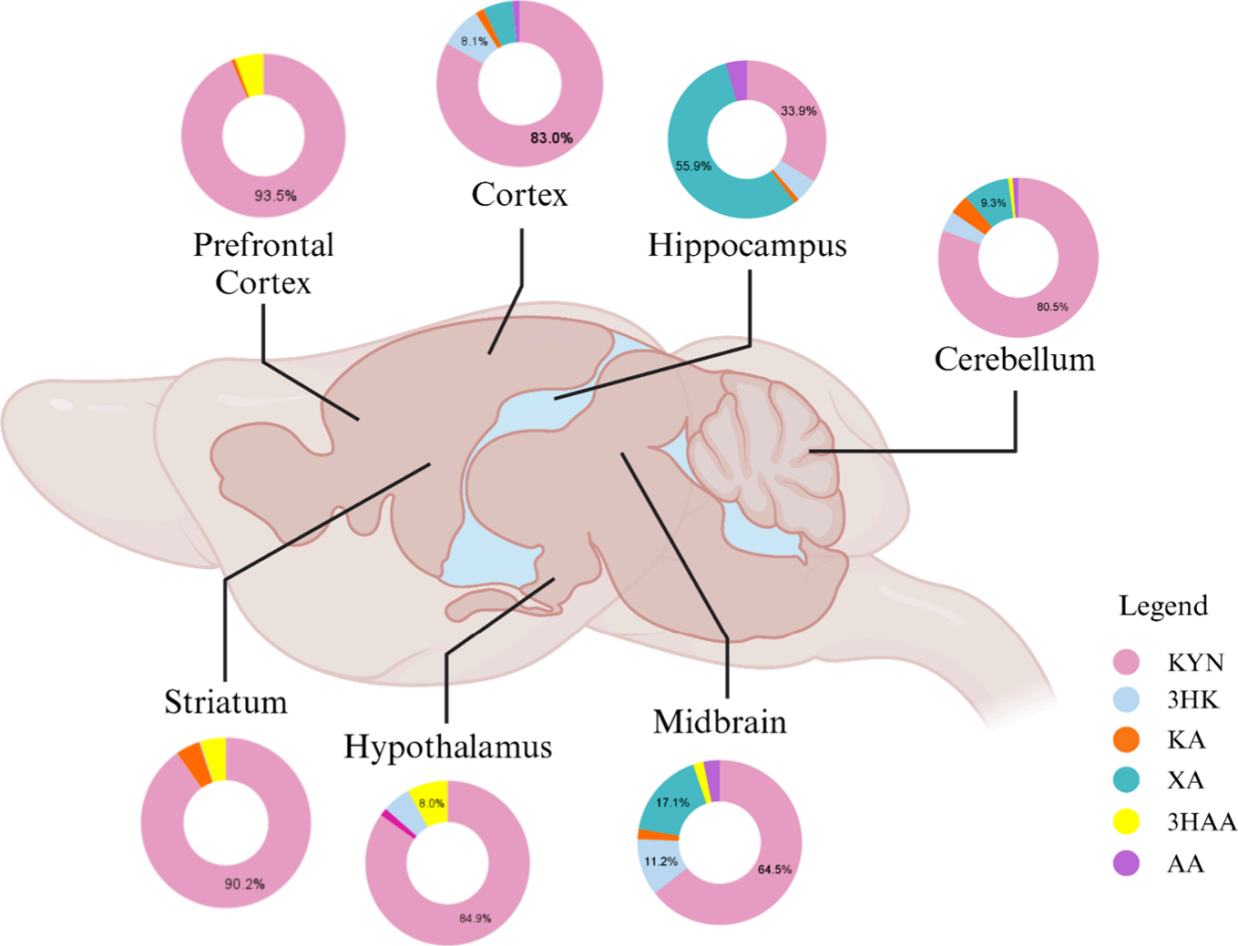
Regional relative abundance of kynurenine and selected metabolites in different brain regions in control rats. The brain regions of interest are highlighted (prefrontal cortex, cortex, hippocampus, cerebellum, striatum, hypothalamus, and midbrain) with donut charts illustrating the relative abundance of kynurenine (KYN), 3-hydroxykynurenine (3HK), kynurenic acid (KA), xanthurenic acid (XA), 3-hydroxyanthranilic acid (3HAA), and anthranilic acid (AA) at basal levels. Results are presented in percentages (*n* = 5). Created with permission from BioRender.com.

### Changes in the Regional Expression of KYN and Its Associated Metabolites following Systemic Administration

#### Acute Administration of KYN

Table 1 shows the plasma pharmacokinetic parameters of KYN for this dosage.

**Table 1:** Plasma Pharmacokinetic Parameters following Administration of a Single Dose of KYN^a^

pharmacokinetic parameters	
*C*_max_ (ng/mL)	24,084
*T*_max_ (h)	0.5
*t*_1/2_ (h)	2.21
elimination rate (L/h)	0.31

a The *C*_max_ concentration is calculated as the average value.

Interestingly, KYN displayed region-specific differences in pharmacokinetic parameters when assessing the tissue distribution of KYN at various time points postdosing. We found that there was a rapid uptake and distribution of KYN in the PFC, COR, STR, HIP, and MDB with all regions reaching *C*_max_ at 30 min postadministration, when compared to basal levels (*p* < 0.05). However, no significant increase was observed in the hypothalamus (HYP) and cerebellum (CER). The PFC showed the highest uptake and accumulation of KYN, reaching a concentration of 87,892.3 ng/g within 30 min, while the hippocampus had the lowest tissue distribution at 9496.2 ng/g (Figure 2). The finding of increased KYN accumulation in the PFC demonstrates the importance of this region’s role in the development of several neuropsychiatric disorders. This increased susceptibility of the PFC to pathophysiological could be attributed to the regions with high density of l-amino acid transporter 1 (LAT1), which increase the uptake of l-amino acids to the region,^22^ and the α7 nicotinic acetylcholine receptor (α7 nAChR)­12 and the *N*-methyl-d-aspartate (NMDA) receptors, which are involved in cholinergic and glutamatergic activity.^1,23^ Increased KYN binding in the PFC may then protect against decreases in acetylcholine and glutamate signaling, which are implicated in the development of neuropsychiatric disorders. The concentrations of KYN metabolites at various time points postadministration are given in Table S2. Baseline levels indicated that the hippocampus had the lowest median KYN concentration at 52.8 ng/g (*n* = 5), comparable to levels reported by Lucchetti et al.^24^ in the control rat hippocampus with a median of 52.0 ng/g (*n* = 9), whereas the striatum had the highest median concentration at 1294.4 ng/g (*n* = 5). The plasma average concentration was 24,084.2 ng/mL at 30 min, with a baseline concentration of 1127.7 ng/mL (*n* = 5), which is nearly two times higher than the baseline values reported in other studies from rat serum with an average of 686 ng/mL (*n* = 6)^25^ and baseline levels in rat plasma with a median of 561 ng/mL (*n* = 5).^24^ Notably, the highest fold change was observed in the hippocampus (FC = 118, *p* = 0.001), while the striatum exhibited the lowest fold change (FC = 11, *p* = 0.011). In comparison, the plasma fold change was intermediate, at 24 (*p* = 0.001). These findings suggest region-specific effects of KYN administration, with significant variations in fold changes and baseline concentrations, which may enhance our understanding of KYN’s role in brain function and its potential therapeutic applications. These results show that KYN and its metabolites have differential uptake and tissue distribution in the various brain regions; this is of significant relevance as it may allow for regional targeted therapy in the application of KYN’s neuroprotective effects.

**Figure 2:**
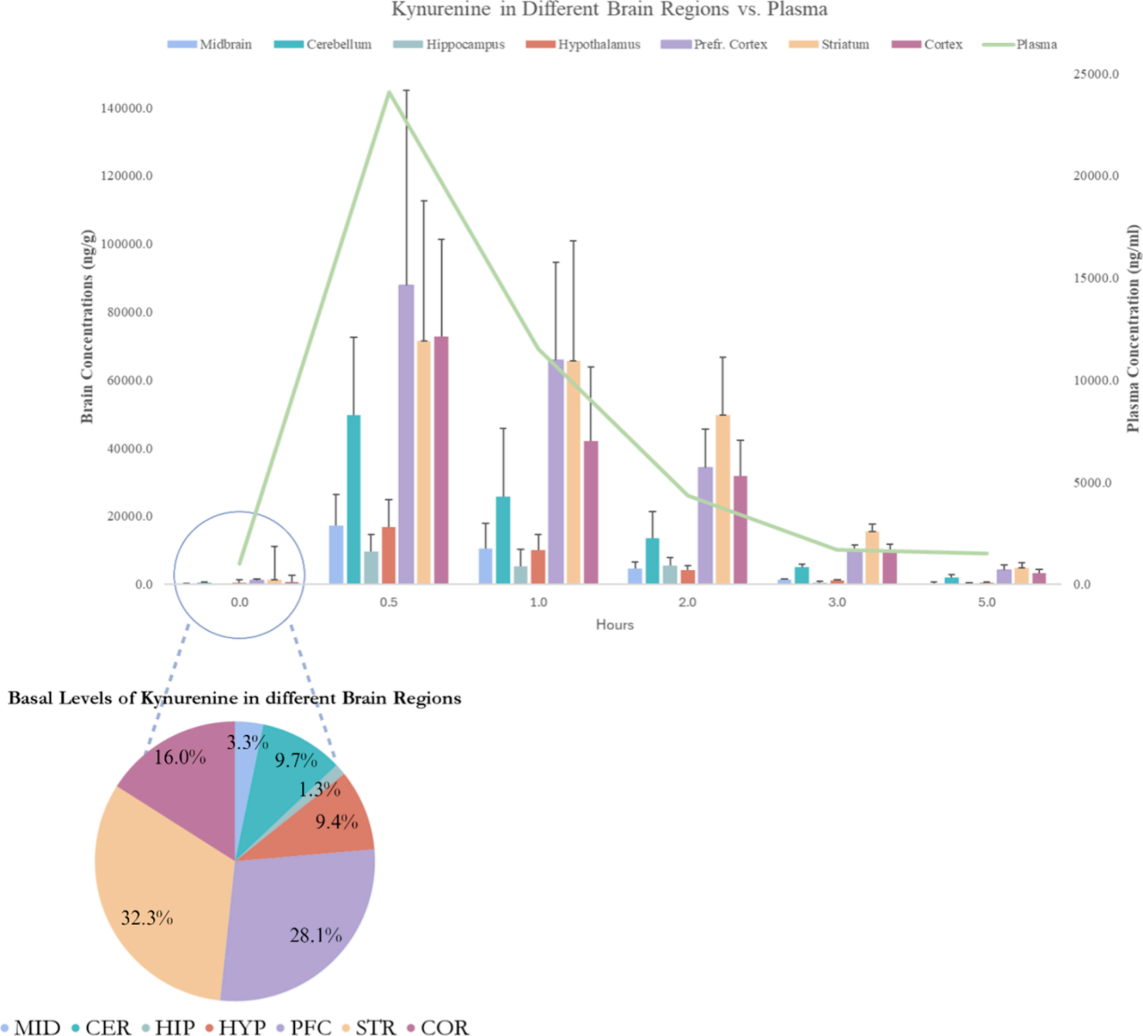
Pharmacokinetics of kynurenine in the rat brain and plasma. The figure includes a combined chart with a column and line graph (top) and a pie chart (bottom left). The column and line charts display time (hours) on the *x*-axis, brain concentration (ng/g) on the left *y*-axis, and plasma concentration (ng/mL) on the right *y*-axis. They present data for various brain regions: the hippocampus (HIP), hypothalamus (HYP), prefrontal cortex (PFC), striatum (STR), midbrain (MID), cerebellum (CER), and cortex (COR). The plasma concentration is represented by a green line. Kynurenine concentrations were measured in rats injected with kynurenine (100 mg/kg, *n* = 25) and were terminated over 5 h and in rats injected with saline (*n* = 5) at 0 h. Results are presented as medians for the brain regions and means for the plasma, with standard deviation of the single measurements as error bars. The kynurenine concentration peaks at 0.5 h in both brain regions and plasma. The prefrontal cortex reached the highest median concentration of 87,892.3 ng/g among brain regions, and the highest plasma concentration was a median of 24,084.2 ng/mL. Both brain and plasma concentrations significantly decrease by 1.0 h and approach the baseline by 5 h. The pie chart shows the kynurenine distribution in different brain regions of saline-injected rats with the results presented as percentages. The striatum had the highest proportion (32.3%), followed by the prefrontal cortex (28.1%), suggesting their key roles in kynurenine metabolism or distribution.

#### Blood–Brain Barrier Permeability

One significant factor to consider is if intraperitoneal KYN administration results in the transport of its metabolites across the blood–brain barrier (BBB), a selectively permeable membrane that regulates the flow of ions and molecules between the blood and the brain.^26^ The BBB is relatively impermeable under normal physiological conditions, but substances like histamine, 5-HT, and free radicals can make it more permeable.^26^ There are two mechanisms by which molecules may cross the BBB, through either active transport or passive diffusion. Reports have shown that TRP, KYN, and to a lesser extent 3HK are actively transported across the BBB via LAT1 transporters.^27^ QA, KA, AA, and 3HAA on the other hand are able to passively diffuse across the BBB.^27^ As a result, it is believed that the cerebral concentration of these metabolites is primarily derived from local production.^27^ We indirectly estimated the BBB permeability of KYN metabolites by analyzing the brain/plasma ratio for each of them. Figure S3 shows box plots visualizing the brain to plasma ratio of both kynurenine and kynurenic acid over time. Half an hour post-KYN administration, the brain/plasma ratio of KYN increased, KYN levels in the brain became higher than plasma concentrations, and the ratio peaked at 2 h postadministration. This demonstrates KYN’s ability to cross the BBB. Similarly, after KYN injection, KA levels in the brain also increased compared to plasma concentrations, indicating either a passive diffusion or increased KYN to KA metabolism in the brain. However, brain KA levels were consistently lower than plasma levels compared to KYN levels; for example, at the 2 h time point, the brain/plasma ratio for KYN was 1.8, compared to 0.54 for KA (Figure S3). Our findings of the elevated KYN brain/plasma ratio, which peaks 2 h after KYN administration, support the hypothesis that intraperitoneally administrated KYN passes the BBB. However, future studies using isotopically labeled KYN would provide more precise evaluation of BBB transport mechanisms.

#### Assessing Neuroprotection

According to research, the KA/QA and KA/3HK ratios may serve as indicators of neuroprotection or neurotoxicity. It has been observed that inflammation can disrupt these ratios.^28^ Therefore, to evaluate the potential neuroprotection after KYN administration, plasma KA/QA ratios were measured (Figure S4) while brain KA/QA ratios were not reported since our approach was unsuccessful to measure brain QA due to the matrix effect. Using a different solvent for protein precipitation during sample preparation or using solid-phase extraction to extract QA will help to measure QA in brain samples with a minimum matrix effect.

There was a significant increase in the plasma KA/QA ratio (*p* < 0.05) of 30 min post-KYN administration and chronically KYN-treated rats compared to their respective saline-treated rats. The acute and chronic study had a 2.5- and 2-fold increase in the KA/QA ratio, respectively. This result suggests a peripheral neuroprotection since KYN therapy raises KA levels relative to QA. For the KA/3HK ratio, both acute and chronic studies demonstrated significant increases in several brain regions of KYN-treated rats. In the acute study, the prefrontal cortex showed a 30-fold increase, while the striatum, hippocampus, and midbrain exhibited 6-fold, 4-fold, and 2-fold increases, respectively. However, there was a 0.3-fold decrease in the plasma KA/3HK ratio, indicating a potential rise in neurotoxic 3HK levels in the periphery. The chronic study showed similar increases in the KA/3HK ratio in the midbrain, hypothalamus, and prefrontal cortex, with fold changes of 6, 6, and 4, respectively. However, the cerebellum showed a significant decrease in the KA/3HK ratio, which may suggest region-specific effects of KYN metabolism and potential neurotoxicity in the cerebellum. As this brain region is essential for motor coordination, balance, and cognitive processes, the cerebellum is particularly vulnerable to the effects of neurotoxicity.^29,30^ In disorders where cerebellar dysfunction is prominent such as ataxia or autism spectrum disorder (ASD), neurotoxicity exacerbates neuronal loss, disrupts cerebellar circuits, and impairs synaptic plasticity.^31,32^ This can lead to worsening of motor deficits, sensory abnormalities, and cognitive dysfunction, compounding the severity of these conditions.

These results indicate a potential peripheral neuroprotection due to an elevated KA/QA ratio after KYN administration, but a reduction of the KA/3HK ratio in region-specific might indicate neurotoxicity. These findings highlight the complex systemic and central effects of kynurenine metabolism, emphasizing the need for further research to understand the balance between neuroprotection and neurotoxicity.

#### Acute and Chronic Administration of KYN

Dysregulation of the kynurenine pathway operates on a continuum from adaptive to pathogenic, contingent on temporal dynamics, cellular context, and enzymatic balance. While acute activation may transiently confer immune or neuroprotective benefits, chronic KP overactivation underlies sustained neurotoxicity and immune dysfunction. Exogenous kynurenine administration, therefore, is a strategy that demands metabolic foresight: beneficial under select circumstances but potentially harmful if the enzymatic milieu is skewed toward toxicity. Therapeutic engagement with KP must navigate these complexities, emphasizing precision modulation over indiscriminate supplementation.

The distribution of KYN and related metabolites in the brain and plasma was analyzed using hierarchical clustering visualized as a heat map (Figure 3), which correlates KYN metabolites with different brain regions and plasma using the Euclidean distance and the Ward algorithm. The baseline distribution of KYN and its metabolites in control rats (*n* = 6) administered saline during the chronic study showed the highest KYN levels in the striatum (Figure 3a). The prefrontal cortex showed the highest concentration of KYN, 30 min post-KYN administration (Figure 3b). In comparison, the striatum displayed the highest concentration of KYN among other brain regions following chronic treatment (Figure 3c) though this increase is not significantly higher than saline levels (Table S3). The average fold change of KYN metabolites in brain regions and plasma 30 min post-KYN treatment compared to saline control levels (Figure 3d) showed a higher fold change of KYN and KA in the hippocampus among other brain regions. This finding suggests that KYN preferentially crosses the BBB and accumulates in the hippocampus after acute treatment. Similarly, the hippocampus showed the highest KYN and KA fold change (84 and 21, respectively, *p*-value < 0.05) in the chronic study (Table S3). For both acute and chronic administration, the hippocampus displayed the largest fold change for KA, suggesting a neuroprotective effect of KYN administration. The hippocampus is strongly associated with the immune system and is particularly susceptible to inflammation-induced metabolic shifts.^33^ Therefore, the heightened sensitivity is partly due to its high density of microglia, which make up 10–15% of the total cell population in the hippocampus compared to the 5–10% in the other brain regions.^33^ Microglia are in charge of modulating neuroinflammation and when activated in the hippocampus amplify IDO activity and promote the synthesis of KYN.^33,34^

**Figure 3:**
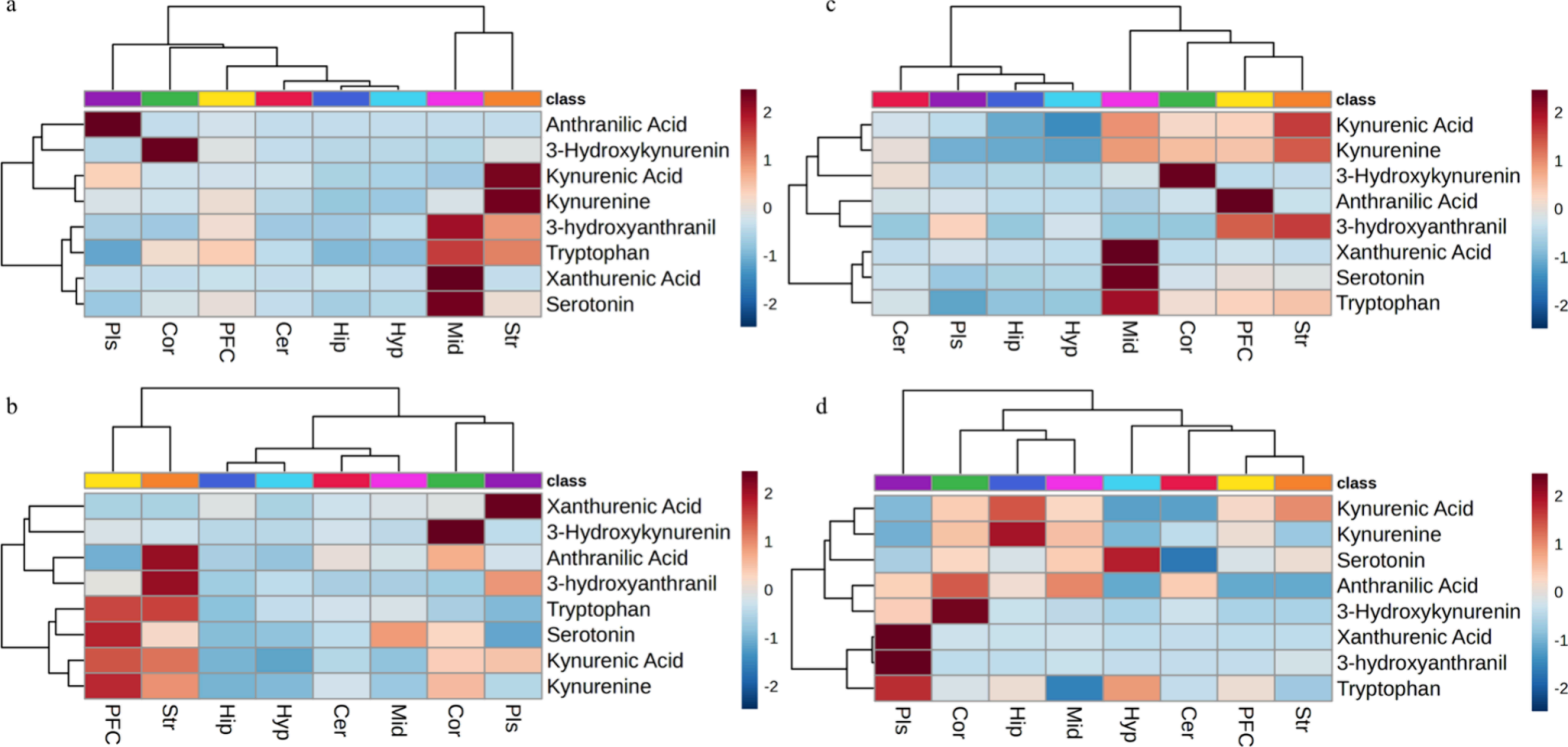
Distribution of kynurenine and related analytes in the brain and plasma. Hierarchical clustering shown as a heat map displays the correlation between kynurenine metabolites and different brain regions or plasma (Pls) (distance measure using Euclidean, algorithm using Ward). Each column represents a brain region or plasma, and each row represents a different metabolite. Color intensity indicates metabolite levels, with red indicating higher levels and blue indicating lower levels. (A) Baseline distribution of kynurenine and its metabolites was assessed in control rats (*n* = 6) administered saline during the chronic study, with the highest kynurenine levels found in the striatum (STR). (B) Distribution was also measured 30 min after a single injection of 100 mg/kg kynurenine in rats (*n* = 5); the peak time for most metabolites with the highest kynurenine levels was found in the prefrontal cortex (PFC). (C) Chronic distribution was evaluated after daily administration of 100 mg/kg kynurenine for 14 days in rats (*n* = 6) with the STR again having the highest levels of KYN. (D) Average fold change of KYN metabolites in different brain regions and plasma following KYN administration at 30 min compared to baseline levels with the highest fold change of KYN observed in the hippocampus (HIP).

The localization of the enzyme kynurenine aminotransferase (KAT) in astrocytes within the hippocampus is likely the main cause of the production and buildup of the majority of KA in this area.^35^ The distribution and function of KAT and KA are implicated in several neurological disorders.^35^ Altered kynurenine metabolism, especially increased neurotoxic activity in the hippocampus, is connected to behavioral abnormalities after peripheral immunological challenges such as inflammation-induced depression in major depressive disorder (MDD) and other mental diseases.^13,28^ Dysregulation of the kynurenine pathway reduces hippocampus volume and prefrontal lobe gray matter density, which are essential for cognition.^28^ Our finding of preferential accumulation of KYN and KA in the hippocampus may help explain the neurobiological causes of cognitive deficits under these conditions, linking disrupted KYN metabolism to hippocampal dysfunction and impaired learning and memory.

#### Molecular and Metabolomic Results

The combined results of the RT-PCR and LC-HRMS analyses for the acute study are shown in Figure 4. The mRNA expression levels of *TDO*, *IDO*, *IL-6*, *CREB*,and *BDNF* (Figure 4and Figure S6) are shown over time within four different brain regions including the hypothalamus, prefrontal cortex, striatum, and hippocampus. Overall, *TDO* expressions were highest in the hypothalamus and lowest in the striatum. *TDO* mRNA expression was not significantly different among the studied brain regions, except for the hypothalamus, where a significant increase was observed 1 h post-KYN administration compared to saline levels. Nonetheless, the presence of any TDO activity in the brain is highly intriguing, considering the little existing knowledge of TDO activity in tissues other than the liver. Some even argue that TDO is only present in the liver.^15^ This makes these findings novel and highly interesting for future research. *IDO* mRNA expression showed no significant changes at any time point following KYN administration compared with saline levels in the studied brain regions. The inflammatory marker *IL-6* mRNA expression was significantly lower in the hippocampus 30 min and 1 h after KYN administration compared to saline treatment and basal levels. Increased neuroprotective KA levels in the same brain area may explain this finding. Overall, the *IL-6* mRNA expression varied within the studied brain regions.

**Figure 4:**
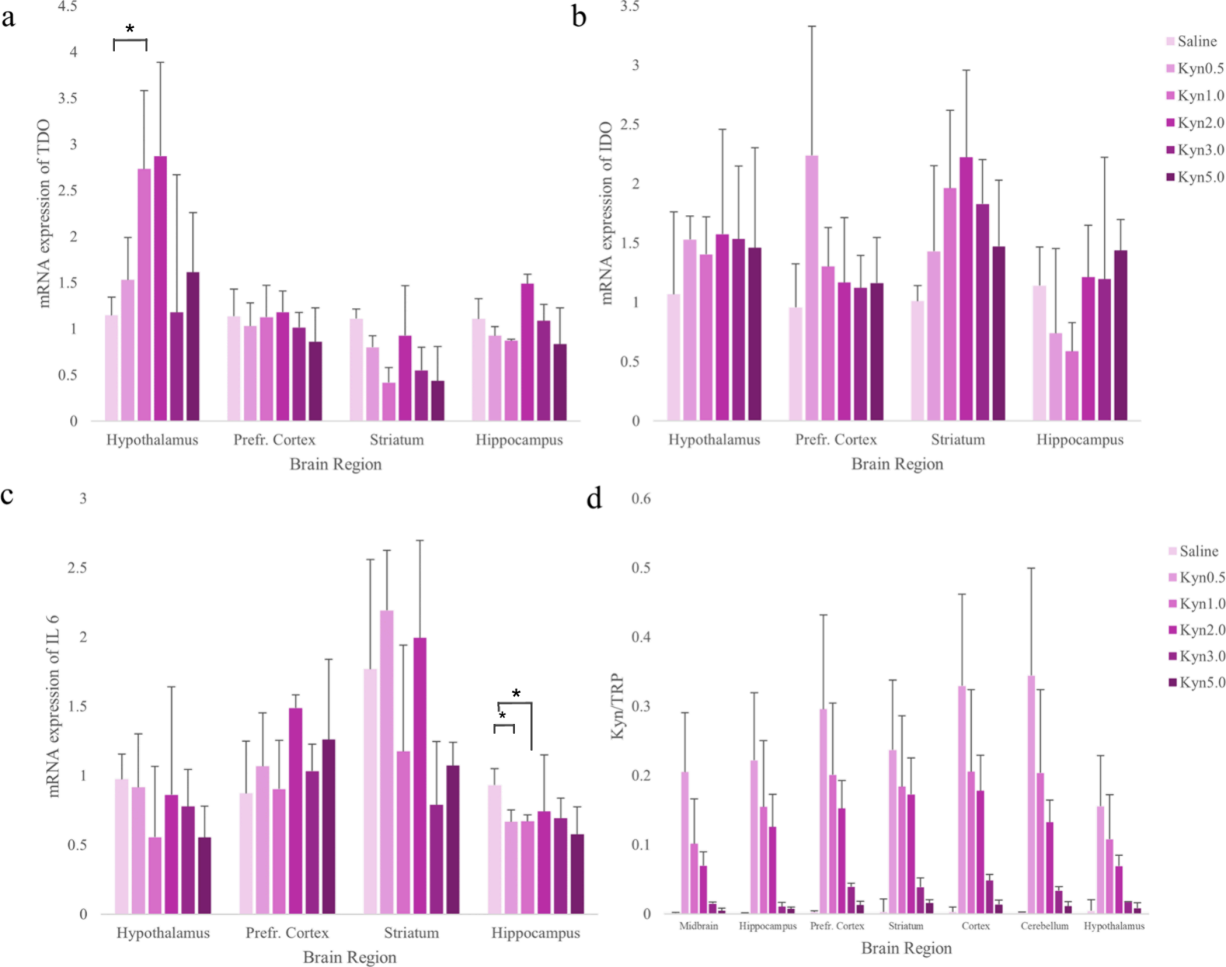
Combined results of the RT-PCR and LC-HRMS analyses for the acute study. The molecular analysis includes mRNA expressions of (a) TDO, (b) IDO, and (c) IL-6 for the hypothalamus, prefrontal cortex (PFC), striatum (STR), and hippocampus (HIP), and the metabolomic analysis results show (d) the kynurenine (KYN)/tryptophan (TRP) ratio within the midbrain (MID), cerebellum (CER), HIP, HYP, PFC, STR, and COR. The results are depicted as columns for each brain region and time point (*n* = 5 rats for each time point) of the acute study, and values reported are medians. The dotted line shows the trend line. Error bars (positive) are given as the standard deviation. Two-sample *t* tests were performed to test for significant differences between saline-treated (saline) and KYN-treated (KYN0.5, KYN1.0, KYN2.0, KYN3.0, and KYN5.0) rats. The tests were performed with the following parameters: normalized data (log transformation and Pareto scaling), parametric, equal group variance, unpaired, and an FDR threshold of 0.05. Significant differences were considered with a *p*-value <0.05 and marked with an asterisk (*). The molecular results showed only significant differences in TDO levels in the hypothalamus between saline and Kyn1.0. IDO showed no significant differences. *IL-6* had a significant decrease in levels in the hippocampus between saline and KYN0.5 and saline to KYN1.0. The results showed significant differences in the KYN/TRP ratio between saline and Kyn0.5 for all brain regions, except for HYP (*p* = 0.28). For KYN1.0 and KYN2.0, all KYN/TRP ratios showed significant differences to the saline treatment. For KYN3.0, all KYN/TRP ratios in the brain regions, except for HYP (*p* = 0.17), were significantly different compared to the saline treatment with the following results: for KYN5.0, only PFC (*p* = 2.2 × 10^–4^), HIP (*p* = 5.4 × 10^–4^), CER (*p* = 0.004), and MID (*p* = 0.02) were significantly different compared to the saline treatment.

The KYN/TRP ratio is shown over time within the seven brain regions (Figure 4d). Here, an increase in the product to substrate ratio can be correlated to an increase in enzyme activity. In theory, it can be used to indirectly estimate enzyme activity levels, but within this study, due to the administration of KYN, it is hard to discern between an increase in KYN due to administration or increased enzyme activity of IDO and TDO to degrade TRP into KYN. This is where the comparison of the KYN/TRP ratio to mRNA expression of *IDO* and *TDO* proves to be helpful. 30 min after KYN administration, the KYN/TRP ratio increased significantly in each brain region. However, when comparing this to the IDO activity levels, there was no significant increase. As such, the increase in the KYN/TRP ratio is not likely due to IDO activity. 1 h after KYN administration, TDO showed a significant increase compared to basal levels in the hypothalamus, indicating that there might be some TDO-induced TRP to KYN metabolism at this time point. Finally, the examined brain areas exhibited no significant change in the mRNA expression of *BDNF* or *CREB* after KYN treatment compared to baseline values (Figure S6). The study found that the hypothalamus had the greatest level of TDO mRNA expression, with a notable rise observed 1 h after KYN administration in the acute study. This suggests that TDO activity may extend beyond the liver to other parts of the brain. However, there were only slight changes in the mRNA expressions of *IDO*, *IL-6*, *BDNF*, and *CREB*. The KYN/TRP ratio revealed an increase in the KYN levels but did not necessarily represent enzyme activity.

The mRNA expression levels of *TDO*, *IDO*, *IL-6*, *CREB*,and *BDNF* and the LC-HRMS levels of KYN/TRP after chronic KYN or saline administration in the hypothalamus, prefrontal cortex, striatum, and hippocampus are depicted in Figure 5and Figure S7. Compared to basal levels, KYN therapy does not significantly modify the *TDO* and *IDO* expression levels in any brain region while there was a considerable rise in *IL-6*’s expression. It has been suggested that *IL-6* exerts both pro- and anti-inflammatory effects in the brain.^36^ Furthermore, *IL-6* plays a critical role in neurogenesis by acting in a neurotrophic capacity.^36^ *IL-6* signals, and its binding subunits on the *IL-6* receptor (*IL-6*R), converge at the gp130/IL6ST homomeric receptor complexes in neurons to initiate Jak/Stat signaling, which are essential for the regenerative action of the nerve growth factor (NGF).^37,38^ Furthermore, when *IL-6* signaling activates the Jak/Stat signaling pathway, it can stimulate the proregenerative actions of neurotrophins such as *NT-3* and *BDNF*, promoting neural repair and recovery.^37,38^ Together, this demonstrates the role of *IL-6* in the region-specific neuroprotective effects of KYN with chronic administration, targeting the prefrontal cortex and the hippocampus. Our findings suggest that KYN exposure in the short term may have anti-inflammatory effects, as *IL-6* expression was decreased. However, in the long term, the mechanisms of action of KYN may be different in that it increases *IL-6* expression, which may serve as a compensatory protective mechanism by increasing neurotrophic pathways due to chronic increases in *IL-6*. Therefore, neurodegenerative disorders such as Alzheimer’s and depression may benefit from KYN treatment through its ability to differentially modulate *IL-6* levels. The dual actions highlight the therapeutic potential of KYN in managing both inflammation and neurodegeneration associated with these conditions.

**Figure 5:**
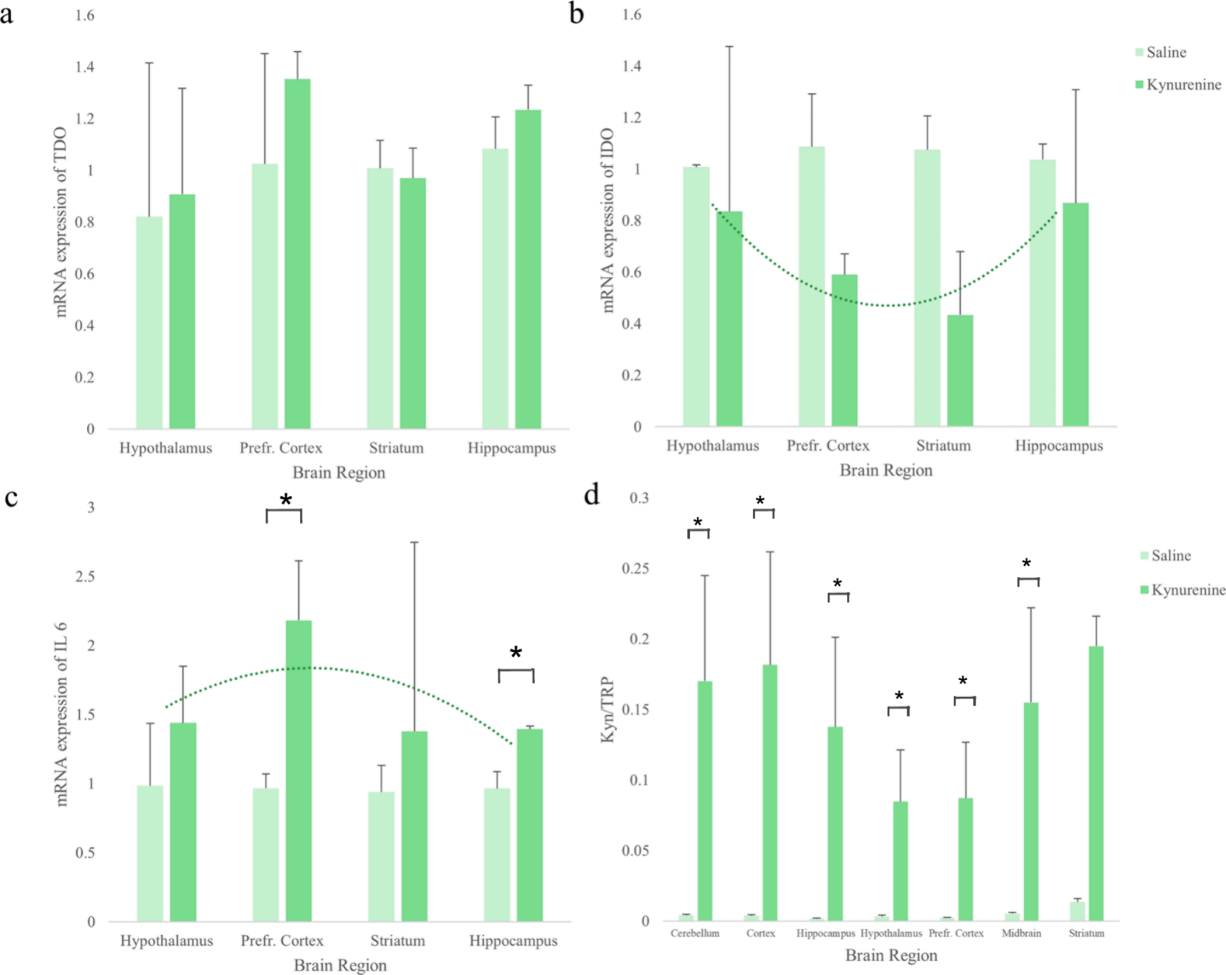
Combined results of the RT-PCR and LC-HRMS analyses for chronic study. The molecular analysis includes mRNA expressions of (a) TDO, (b) IDO, and (c) IL-6 for the hypothalamus (HYP), prefrontal cortex (PFC), striatum (STR), and hippocampus (HIP), and the metabolomic analysis results show (d) the kynurenine (KYN)/tryptophan (TRP) ratio within the midbrain (MID), cerebellum (CER), HIP, HYP, PFC, STR, and COR. The results are depicted as columns for each brain region, showing the results of saline- and kynurenine-treated rats. The dotted line shows the trend line. Error bars (positive) are given as the average standard deviation of the brain regions. Two-sample *t* tests were performed on the results to test for significant differences between the saline-treated (saline) and KYN-treated (kynurenine) rats. The tests were performed with the following parameters: normalized data (log transformation and Pareto scaling), parametric, equal group variance, unpaired, and an FDR threshold of 0.05. Significant differences were considered with a *p*-value < 0.05. TDO and IDO showed no significant differences in any brain region between saline and kynurenine treatment. IL-6 showed significant differences for the PFC (*p* = 0.008) and HIP (*p* = 0.02). Trendlines in (b) and (c) showcase interesting opposing concentration trends between IDO and IL-6, where IDO levels decreased in the prefrontal cortex and striatum and IL-6 levels increased within these brain regions (mostly the PFC). The metabolomic results showed significant differences in the KYN/TRP ratio between saline and kynurenine treatment for all brain regions, except for STR (*p* = 0.25).

The seven brain areas in Figure 5d illustrate the KYN/TRP ratio following chronic kynurenine or saline treatment. KYN was dramatically boosted in all brain areas except the striatum. The increases are presumably attributable to kynurenine, not to enzyme activity. Finally, *CREB* and *BDNF* showed no significant differences in any brain region between saline and kynurenine treatment (Figure S7). To sum up, chronic KYN administration led to an increases in *IL-6* expression in the prefrontal cortex and hippocampus, potentially promoting neuroprotection through neurotrophic pathways. This suggests that KYN may have therapeutic potential for neurodegenerative disorders like Alzheimer’s and depression by modulating *IL-6* levels to balance inflammation and neurodegeneration.

## Methods

### Study Design

The dose of kynurenine and the number of rats were selected based on previous studies in the field, which investigated the effect of kynurenine administration in preclinical models.^25,39–45^

### Ethical Approval

All procedures were in accordance with the Animal Ethics and Control Committee of the University of the Witwatersrand animal care regulations and were approved by the Institutional Animal Ethics Screening Committee of the University of the Witwatersrand (approval reference no. AREC23-03-009).

### Animals

Male Sprague–Dawley rats (the average animal weights were 205 g for the saline and 225 g, *n*_total_ = 42) were housed two per cage at an ambient temperature of 25 ± 2 °C, on a 12 h light/dark cycle (lights on 07h00). Standard laboratory rodent feed (Lab Chef, Johannesburg, South Africa) and normal drinking water were provided *ad libitum.*

### Drug Preparation

The following drugs were used in this study: saline (Adcock Ingram, Johannesburg, South Africa) and l-kynurenine (Cayman Chemical Company, Ann Arbor, Michigan, item no. 11305), which was prepared in saline. Drugs were freshly prepared and injected in a volume of 0.2 mL of kynurenine (ip) and 0.2 mL of saline (ip) per rat.

### Acute Study

For the acute study, experimental animals were randomly divided into two groups: control (*n* = 5) and kynurenine-treated (*n* = 25). Rats received either a single dose of kynurenine (100 mg/kg, *n* = 25) or saline (*n* = 5) via intraperitoneal (ip) injections. The kynurenine-treated animals received 100 mg/kg, ip at *t*_0_, and were terminated at 0.5, 1, 2, 3, and 5 h postkynurenine administration (*n* = 5 per time point). These time points were selected based on the rapid metabolism of KYN and its short half-life, allowing us to track changes in metabolite concentration following a single dose.

### Chronic Study

For the chronic study, rats were randomly divided into two groups: control (*n* = 6) and kynurenine-treated (*n* = 6). Rats were injected intraperitoneally (ip) with 100 mg/kg kynurenine (*n* = 6) and saline (*n* = 6) once a day for 14 days to allow for the bioaccumulation of KYN in tissues and for a steady state to be reached. A 14-day duration was chosen as previous studies have shown that KYN reaches steady state concentration at 90 min post a single dose,^1^ suggesting that daily doses for 14 days would be sufficient to reach stable plasma and brain concentrations. In addition, it has been shown that following 5 days of a lower dose of kynurenine (75 mg/kg) administration, there are significant antioxidant effects in the brain.^2^ Therefore, 14 days of kynurenine treatment is sufficient to achieve stable plasma and tissue drug concentrations and to achieve pharmacodynamics efficacy. Rats were then terminated 1 h post the last KYN injections by decapitation using a rodent guillotine.

### Tissue Collection

Following decapitation*,* rodent brains were surgically removed, and the following brain regions were dissected on ice: HYP, PFC, STR, HIP, MID, COR, and CER. Plasma, visceral adipose tissue, the spleen, and liver were also harvested for analysis. All tissue samples were weighed, flash-frozen in liquid nitrogen, and stored at −80 °C until molecular analysis.

### Molecular Analysis

#### Chemicals and Reagents

Our method contained 15 analytes. AA, biotin (B7), 3HAA, 3HK, KA, phenylalanine (PhA), pyridoxal 5′ phosphate (PLP), riboflavin (B2), 5-HT, TRP, tyrosine (TYR), KYN, and XA were purchased from Sigma-Aldrich. QA was obtained from Tocris Bioscience and pantothenic acid (B5) from the European Pharmacopoeia Reference Standard (Strasbourg, France). Stable isotope internal standards AA-[^13^C_6_], 3HAA-[^13^C_6_], 3HK-[^13^C_6_], KYN-[^13^C_6_], KA-*d*_5_, QA-[^13^C_4_-^15^N], 5-HT-*d*_4_, theobromine, TRP-[^13^C_11_-^15^N_2_], and XA-[^13^C_6_] were purchased from Alsachim (Illkirch-Graffenstaden, France). B7-*d*_2_ and PhA-*d*_5_ were bought from Sigma-Aldrich, and TYR-*d*_7_ was from Cambridge Isotopes Laboratories (Tewksbury, MA, USA). A Milli-Q water purification system (Millipore, Bedford, MA, USA) was used to purify deionized water (MQ) (18.2 M cm). MeOH, ACN, and IPA were purchased from Fisher Scientific (MA, USA). Merck (Darmstadt, Germany) supplied a 25% ammonia (NH_3_) solution and Sigma-Aldrich formic acid (FA). While the other reagents were at room temperature, MeOH and FA were held at 4 °C.

### Liquid Chromatography High-Resolution Mass Spectrometry

#### Sample Preparation

##### Plasma Samples

Plasma samples were prepared as previously described.^46^ Briefly, plasma samples were defrosted, centrifuged, and subjected to protein precipitation by adding an IS mixture, 0.1% FA in MQ water, and cold MeOH. Samples were then vortexed and stored at −80 °C. Following centrifugation, the supernatant was evaporated using a SpeedVac/nitrogen stream and reconstituted in 0.1% FA in MQ water for injection into the liquid chromatography system.

#### Brain Samples

Brain tissue samples were homogenized by adding 0.1% FA in MQ water, MeOH, and homogenization beads (Lysing Matrix D) in a 1:4 (m/v) ratio of the tissue weight to solvent. After vortexing at 1600 rpm for 15 s, samples were homogenized using the FastPrep-24 5G for 3 × 30 s at 6 m/s. Then, 25 μL of the IS mixture was added to half of the homogenized brain extract, and samples were evaporated for 1–3 h using a SpeedVac/N_2_ stream. Throughout homogenization and sample preparation, tissue samples and extracts were kept cooled on dry ice or cooling trays to prevent any postmortem degradation of the analytes of interest.

#### Chromatographic Separation and Mass Spectrometry Analysis

Our earlier analytical method was employed with a few minor adjustments, such as a shorter gradient for the LC method and a reduction in the number of included analytes to 15. For more complete details, see ref 46. Briefly, a Waters Acquity UHPLC was coupled to a high-resolution Q Exactive hybrid quadrupole-Orbitrap mass spectrometer, with heated electrospray ionization. Chromatographic separation was achieved using a Waters HSS T3 column (1.8 μm, 2.1 × 100 mm) maintained at 30 °C. The mobile phases consisted of mobile phase A, Milli-Q water with 0.6% formic acid (100:0.6%, v/v), and mobile phase B, methanol with 0.6% formic acid (100:0.6%, v/v). The LC method was shortened from 10 to 6.5 min using the following gradient: 1% B (initial), 1–60% B (0.3–3 min), 60–90% B (3–3.5 min), hold at 90% B (3.5–4.5 min), 90–1% B (4.5–4.8 min), and 1% B (4.8–6.5 min) for column equilibration. A timed PRM method was used with an inclusion list for the 15 analytes with their respective internal standard (Table S1), and positive ionization mode was used for quantification. Exemplary extracted ion chromatograms are shown in (Figure S2) for all analytes researched during this study.

#### Gene Expression Analysis

##### Sample Preparation

For molecular analysis, brain samples were homogenized using a probe sonicator (Fisher Scientific, Waltham, Massachusetts, USA) whereafter an Illustra minispin RNA extraction kit (GE Healthcare, USA) was used to extract RNA. A SuperScript VILO cDNA synthesis kit (Thermo Fisher, California, USA) was used to synthesize cDNA. A spectrophotometer (NanoDrop One^C^, Thermo Scientific, Waltham, MA, USA) was used to quantify RNA and cDNA.

#### RT-PCR Analysis

Real-time PCR was carried out to determine mRNA expressions of *IDO, TDO*, *BDNF, CREB*, and *IL-6* using RT-PCR TaqMan assays (Thermo Scientific, Waltham, MA, USA), with *Tbp* as the endogenous control. A StepOne real-time PCR system (Applied Biosystems, Foster City, CA, USA) was used to amplify the genes of interest. The 2^–ΔΔCT^ method was used to determine the changes in expression of mRNA and is represented as a fold change relative to the control (Livak and Schmittgen, 2001). The TaqMan assay IDs of the probes used were as follows: *Tbp* (Rn01455646_m1), *IDO* (Rn01482210_m1), *TDO* (Rn00574499_m1), *BDNF* (Rn02531967_s1), *CREB* (Rn01451883_m1), and *IL-6* (Rn01410330_m1).

#### Data Processing and Statistical Analysis

LC-HRMS data acquisition and processing were carried out using Xcalibur software (version 4.7, Thermo Scientific), with tuning and optimization of analytes performed via Tune interface software (Tune v2.9). Stata Software (version Stata/SE 18.0, StataCorp LLC) was used to examine a variety of pharmacokinetic parameters including the elimination rate (ER) and half-life. MetaboAnalyst (version 6.0) and Minitab (version 21.4.2, Minitab, Inc.) were used to evaluate the results and perform statistical analyses. Data normalization was performed via log transformation and Pareto scaling, and data are presented as the median and interquartile range (IQR). Significance testing was performed with either two-sample *t* test for normally distributed data, or the Wilcoxon rank-sum test for non-normally distributed data. A *p*-value threshold (raw) of 0.05 was applied. For the figures, significant differences were marked with an asterisks (*). Heat maps were created with the original data, Euclidean distance measure, Ward clustering, and autoscaling features as standardization. A volcano plot was used for fold change analysis, and nonparametric tests were used. A threshold of two was applied to the ratio of kynurenine to saline.

Google Sheets, Microsoft Excel for Microsoft 365 MSO (version 2406), and BioRender (2024) were used for data visualization. All brain concentrations were presented as median values and the plasma as the average of two separate sample preparations. Standard deviations were used as error bars for the column charts.

## Conclusions

In this study, we assessed the plasma pharmacokinetics of KYN following acute 100 mg/kg administration and determined basal levels of KYN and its metabolites in plasma and seven brain regions. Acute and chronic KYN administration dramatically enhanced the plasma KA/QA ratio, suggesting peripheral neuroprotection. In KYN-treated rats, the KA/3HK ratio increased dramatically in the prefrontal cortex, striatum, hippocampus, hypothalamus, and midbrain, also indicating neuroprotective benefits. However, plasma and cerebellar KA/3HK ratio decreases suggest region-specific neurotoxicity. The hippocampus accumulated the most KYN and its metabolite KA following acute and chronic treatment, suggesting neuroprotective effects. Both RT-PCR and LC-HRMS analyses revealed that TDO levels were highest in the hypothalamus and unchanged across brain regions, except for a significant increase in the hypothalamus 1 h post-KYN administration. IDO levels remained stable, while *IL-6* significantly decreased in the hippocampus at 30 min and 1 h post-KYN administration, correlating with increased KA levels. The KYN/TRP ratio increased significantly in all brain regions, independent of IDO activity, with a significant TDO increase in the hypothalamus at 1 h. No changes were observed in the *BDNF* and *CREB* expression levels.

A limitation of our study is the absence of neurobehavioral testing, which would provide a more comprehensive understanding of the functional impact of KYN administration and strengthen the justification for peripheral neuroprotection beyond relying solely on the KA/QA ratio. Additionally, our reliance on a rat model assumes that the metabolic response to KYN administration closely mimics that of humans, which may not fully capture the complexities of human metabolism. Future studies should investigate the effect of KYN administration in female rats, as sex differences in immune and metabolic responses may influence the outcomes of KYN administration. Moreover, future studies should incorporate diseased rat models to infer disease-related neurological pathways and examine whether varying doses of KYN elicit differential effects, which is crucial to better understand its threshold, efficacy, and potential toxicity. Furthermore, our method was not successful in measuring QA in brain regions, limiting our ability to fully assess the neurochemical changes associated with KYN administration.

## Supplementary Material



## Notes

Tryptophan metabolism pathway, instrumental parameters, extracted ion chromatograms, analyte concentrations in the acute study, box plots of kynurenine brain to plasma ratios, box plots of kynurenic acid to quinolinic acid ratios in plasma, analyte concentrations in the chronic study, and results of the gene expression analysis (PDF)
