# Supplementary material for: Potential Region-Specific Neuroprotective Effects of Kynurenine Administration in Healthy Rodents Using High-Resolution Mass Spectrometry
Source: ACS Chem Neurosci. 2025 Sep 15;16(19):3682–93. doi: 10.1021/acschemneuro.4c00586 (PMC12498425; doi:10.1021/acschemneuro.4c00586)
Supplement: Supplementary file 1 [file cn4c00586_si_001.pdf]

Supporting Information

Potential neuroprotective effects of kynurenine administration in healthy rodents using high resolution mass spectrometry

Sandy Abujrais[a,b], Anne Simeit[a], Mara Link[a], Fleur Kalberg[a], Leandrie Pienaar[c,d], Radhini Veerappan[c,d], Aletta ME Millen[c,d], Sooraj Baijnath[c,d], Jonas Bergquist[a,b]

a Analytical Chemistry and Neurochemistry, Department of Chemistry – BMC, Box 599, Uppsala University, 75124, Uppsala, Sweden

b The ME/CFS Collaborative Research Centre at Uppsala University, Sweden

c Wits Integrated Molecular Physiology Research Initiative, Wits Health Consortium (PTY) Ltd, School of Physiology, Faculty of Health Sciences, University of The Witwatersrand, Johannesburg, South Africa.

d School of Physiology, Faculty of Health Sciences, University of The Witwatersrand, Johannesburg, South Africa.

Corresponding author email address: jonas.bergquist@kemi.uu.se

S1. Background Information

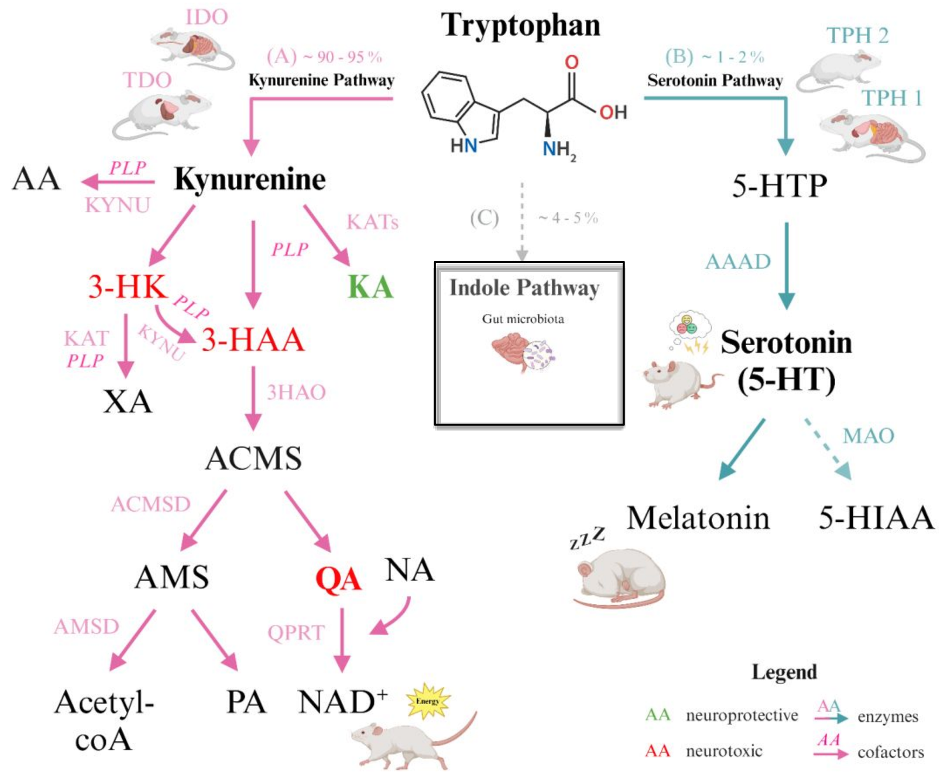

Figure S 1. Tryptophan Metabolism via Kynurenine, Serotonin, and Indole Pathway. (A) ~ 90 % of the TRP degradation to kynurenine occurs in the liver via TDO and the rest occurs via IDO in multiple other tissues, including the brain and GI tract. Kynurenine is degraded into the neuroprotective metabolite KA by KAT enzymes (and cofactor PLP), AA by KYNU (and cofactor PLP) and the two neurotoxic metabolites 3HK by KMO (and cofactor B2) and 3HAA. 3HK is also metabolized into 3HAA by KYNU (and cofactor PLP). 3HAA is further metabolized into ACMS by 3-HAO, which is metabolized via one branch into AMS (by ACMSD), PA and Acetyl-CoA (by AMSD) and another branch into neurotoxic QA, and finally via the Nam catabolism and NA into NAD+ by QPRT. (B) In the serotonin pathway, TRP is metabolized into first 5-HTP via TPH enzymes, then to the neurotransmitter 5-HT via AAAD and finally into melatonin or 5-HIAA via MAO. (C) TRP that was not absorbed is metabolized into several indole derivatives and tryptamine by gut bacteria.

Abbreviations: TDO – Tryptophan 2,3-dioxygenase; IDO – Indoleamine 2,3-dioxygenase; GI tract – Gastrointestinal tract; KA – Kynurenic Acid; KAT – Kynurenine Aminotransferase; PLP – Pyridocal 5'-phasphate; AA – Anthranilic Acid; KYNU – Kynureninase; 3HK – 3-Hydroxykynurenine; KMO - Kynurenine Monooxygenase; B2 – Riboflavin (Vitamin B2); 3HAA – 3-Hydroxyanthranilic Acid; 3-HAO - 3-Hydroxyanthranilic Acid 3,4-dioxygenase; ACMS – 2-Amino-3-Carboxymuconic Acid-6-Semialdehyde; AMS – Aminomuconate-Semialdehyde; ACMSD - 2-Amino-3-Carboxymuconate-Semialdehyde Decarboxylase; PA – Picolinic Acid; Acetyl CoA – Acetyl Coenzyme A; AMSD – 2-Aminomuconic Semialdehyde Dehydrogenase; QA – Quinolinic Acid; Nam – Nicotinamide; NA – Nicotinic Acid; NAD – Nicotinamide Adenine Dinucleotide; QPRT – Quinolinate Phosphoribosyltransferase; 5-HTP – 5-Hydroxytryptophan; TPH 1/2 – Tryptophan Hydroxylase 1/2; 5-HT – 5-Hydroxytryptamine; AAAD – Aromatic Amino Acid Decarboxylase; 5-HIAA – 5-Hydroxyindole Acetic Acid; MAO – Monoamine Oxidase. (adapted from [3,5–7] and created with BioRender.com).

S2. Experimental

Table S1. Instrumental parameters of the applied timed-PRM method showing normalized collision energy (NCE%) for each metabolite and its isotope labeled internal standard. If there are two ions, the underlined product ion is used as the quantifying ion.

ID	Analytes	Abbreviation	Precursor Ion (m/z) [M+H]+	RT (min)	Analysis Range (min)	(N)CE (%)	Product ions (m/z)
1	Quinolinic acid	QA	168.02913	2.23	1.40 - 2.40	60	96.04451
							124.03922
2	Quinolinic acid-[13C4.15N]	QA-[13C4.15N]	173.07834	2.23	1.40 - 2.40	60	99.0486
3	Pyridoxal 5′-phosphate	PLP	248.03185	2.30	1.40 - 2.40	40	150.05495
4	Pyridoxine-[2H3]	B6-[2H3]	173.09945	2.24	1.40 - 2.40	40	155.08949
5	3-Hydroxykynurenine	3HK	225.08698	2.40	1.80 - 2.43	15	208.06007
							110.06012
6	3-Hydroxykynurenine-[13C6]	3HK-[13C6]	231.17676	2.40	1.80 - 2.43	15	214.08353
7	Tyrosine	TYR	182.08117	2.55	2.00 - 2.70	30	136.07563
							165.05466
8	Tyrosine-[2H7]	TYR-[2H7]	189.12511	2.51	2.00 - 2.70	30	143.11952
9	Serotonin	SER	177.10224	2.66	2.00 – 2.70	10	160.07578
10	Serotonin-[2H4]	SER-[2H4]	181.12735	2.66	2.00 – 2.70	10	164.10089
11	Kynurenine	KYN	209.09207	2.97	2.20 - 3.00	20	192.06512
							94.06545

12	Kynurenine-[13C6]	KYN-[13C6]	215.17736	2.97	2.20 - 3.00	20	198.08536
13	Phenylalanine	PhA	166.08626	3.16	2.40 - 3.20	55	120.08096
							90.94795
14	Phenylalanine-[2H5]	PhA-[2H5]	171.11764	3.17	2.40 - 3.20	55	125.11234
15	Pantothenic acid	B5	220.11795	3.15	2.40 - 3.20	10	90.05515
16	Theobromine	The	181.072	3.25	2.40 – 4.00	40	138.06619
							110.07127
17	3-Hydroxyanthranilic Acid	3HAA	154.04987	3.33	2.40 – 3.20	20	136.03903
							108.04455
18	3-Hydroxyanthranilic Acid –[13C6]	3HAA –[13C6]	160.07000	3.33	2.40 – 3.20	20	142.05916
19	Tryptophan	TRP	205.09715	3.62	2.80 – 3.50	10	188.07034
							146.06006
20	Tryptophan-[13C11.15N2]	TRP-[13C11.15N2]	218.12813	3.62	2.80 – 3.50	10	200.10432
21	Xanthurenic acid	XA	206.04478	4.01	3.20 - 4.00	55	178.04951
							132.04419
22	Xanthurenic acid-[13C6]	XA-[13C6]	212.0649	4.01	3.20 - 4.00	55	184.06964
23	Kynurenic acid	KA	190.04987	4.04	3.20 - 4.00	55	162.05472
24	Kynurenic acid-[2H5]	KA-[2H5]	195.08125	4.02	3.20 - 4.00	55	167.08607
25	Biotin	B7	245.09544	4.00	3.20 - 4.00	35	227.0854
26	Biotin-[2H2]	B7-[2H2]	247.10745	4.00	3.20 - 4.00	35	229.09794
27	Riboflavin	B2	377.14556	4.05	3.20 - 4.00	40	243.08792
28	Anthranilic acid	AA	138.05496	4.23	3.20 - 4.00	10	120.04446
							92.04964
29	Anthranilic acid-[13C6]	AA-[13C6]	144.0751	4.23	3.20 - 4.00	10	126.06459

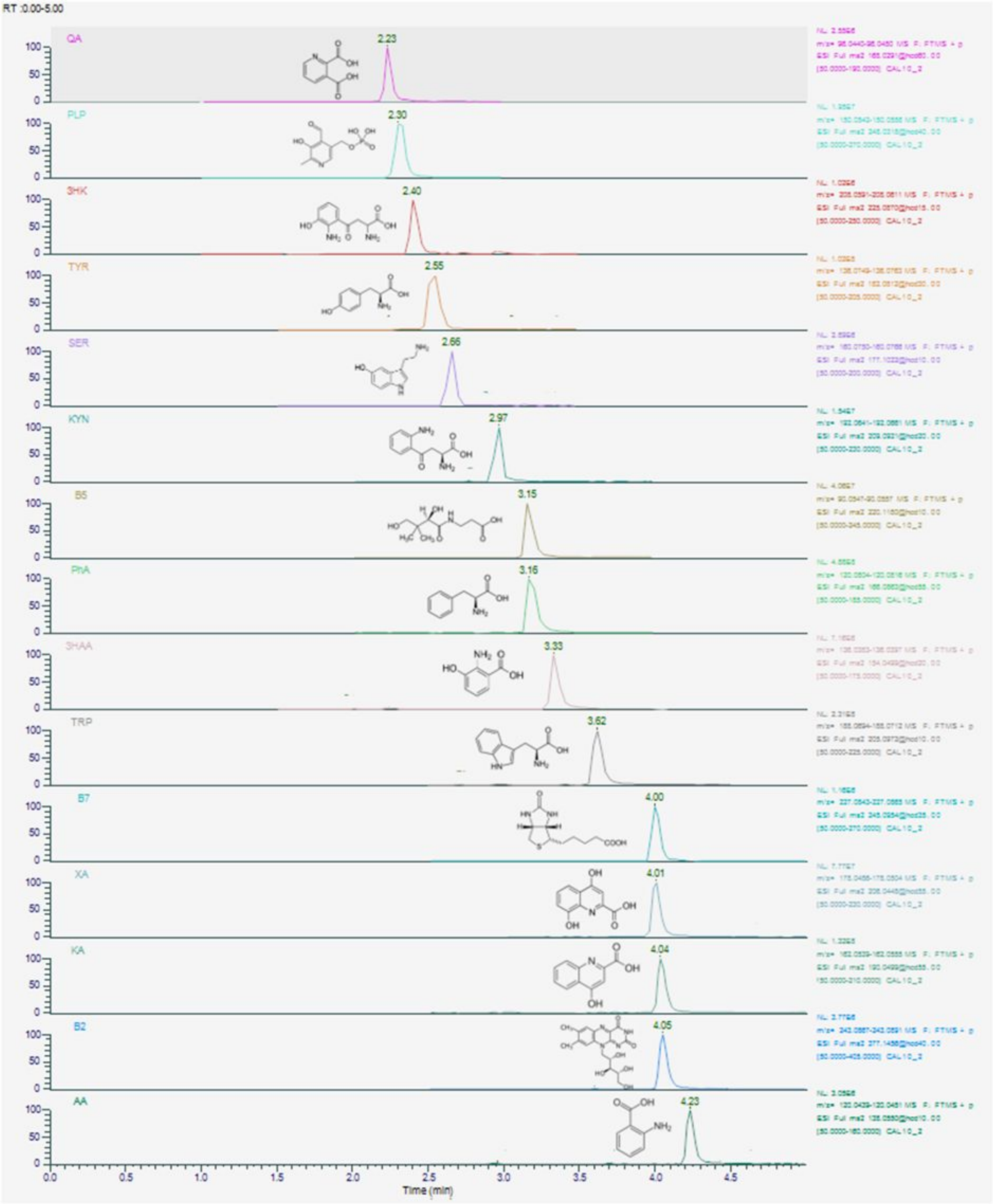

Figure S2. Extracted Ion Chromatograms of 15 analytes in calibrator 10 in plasma analyzed with the UHPLC QqOrbi MS method. Created with Freestyle^{TM}1.8 SP2 QF1.

S3. Results

Acute Study

Table S2. Analyte concentrations in the acute study. Results include 7 brain region concentrations (ng/g), with the median value reported (n=5 for PFC and STR; n=10 for MID, CER, HIP, HYP and COR), overall brain median concentration (ng/g), and plasma concentration (n=10) (ng/ml), which represents the average of two sample preparations.

Tryptophan									
Hours	MID	CER	HIP	HYP	PFC	STR	COR	Median	Plasma
0.0	109149.3	174737.3	60945.7	90642.3	365644.5	504073.4	225858.6	174737.3	6511.4
0.5	100507.4	142325.0	54255.2	96252.9	328559.1	345096.4	214387.5	142325.0	9561.7
1.0	88926.2	150483.3	44481.7	81337.3	308869.4	325630.6	192493.8	150483.3	9232.5
2.0	81033.8	129309.9	47534.6	62410.9	247427.4	339897.2	185189.6	129309.9	9269.3
3.0	78805.7	150279.2	42610.5	68464.9	260735.8	306733.6	192174.2	150279.2	9309.6
5.0	72462.1	150075.6	48843.3	53167.1	310460.8	339294.3	184713.4	150075.6	7892.2
Serotonin									
Hours	MID	CER	HIP	HYP	PFC	STR	COR	Median	Plasma
0.0	4463.8	3271.0	960.3	940.6	9299.8	4468.3	4016.1	4016.1	341.0
0.5	5359.5	2407.3	915.7	1225.6	8254.7	2950.6	3742.9	2950.6	213.5
1.0	3633.1	3234.9	787.5	3168.1	6710.0	3441.2	3618.8	3441.2	478.4
2.0	3892.2	3616.9	973.6	1486.1	7472.9	4687.1	3624.9	3624.9	315.2
3.0	4159.3	3414.0	1035.8	3287.2	7571.5	3334.1	4018.7	3414.0	314.6
5.0	3961.6	4014.6	1237.7	1678.6	10189.5	5442.7	4170.9	4014.6	348.0
Kynurenine									
Hours	MID	CER	HIP	HYP	PFC	STR	COR	Median	Plasma
0.0	130.3	387.5	52.8	375.4	1123.3	1294.4	640.1	387.5	987.5
0.5	17260.6	49629.0	9496.2	16742.4	87892.3	71516.8	72807.9	49629.0	24084.2
1.0	10460.6	25665.1	5223.6	10021.6	65849.2	65540.9	42066.3	25665.1	11523.3
2.0	4530.1	13447.7	5449.0	4080.2	34184.6	49581.2	31844.2	13447.7	4338.8
3.0	1217.8	5023.5	475.4	1188.0	9493.7	15434.2	9406.1	5023.5	1682.3
5.0	316.8	1997.7	317.6	491.1	4059.0	4757.1	3305.1	1997.7	1504.9
Kynurenic Acid									
Hours	MID	CER	HIP	HYP	PFC	STR	COR	Median	Plasma
0.0	4.1	19.2	1.6	6.7	9.7	66.7	12.8	46.5	9.7
0.5	166.4	346.5	105.7	66.9	936.8	716.4	560.4	487.6	346.5
1.0	99.2	208.0	85.6	71.2	518.0	653.1	338.5	211.7	208.0
2.0	55.7	99.1	51.0	12.7	285.0	464.7	225.4	135.7	99.1

3.0	17.5	46.6	19.2	6.4	129.0	219.2	80.7	65.2	46.6
5.0	5.3	20.6	7.9	6.8	31.0	90.4	31.6	49.7	20.6
Anthranilic Acid									
Hours	MID	CER	HIP	HYP	PFC	STR	COR	Median	Plasma
0.0	6.5	5.5	6.8	<LOQ	<LOQ	<LOQ	11.1	6.6	10.3
0.5	88.2	143.9	37.0	<LOQ	<LOQ	<LOQ	171.5	116.1	70.0
1.0	26.3	28.6	10.2	<LOQ	<LOQ	<LOQ	37.5	27.4	25.3
2.0	5.8	17.2	5.6	<LOQ	<LOQ	<LOQ	14.6	10.2	12.4
3.0	3.6	14.6	5.4	<LOQ	<LOQ	<LOQ	16.7	10.0	10.2
5.0	3.1	4.2	5.7	<LOQ	<LOQ	<LOQ	10.9	5.0	9.4
3-Hydroxykynurenine									
Hours	MID	CER	HIP	HYP	PFC	STR	COR	Median	Plasma
0.0	22.5	19.9	7.5	<LOQ	<LOQ	<LOQ	62.5	21.2	7.9
0.5	375.1	1131.2	175.2	307.0	1408.7	1289.5	4600.8	1131.2	513.7
1.0	519.8	5541.4	182.7	336.7	2057.5	1904.4	19105.1	1904.4	205.1
2.0	334.0	15604.0	243.6	343.5	1926.8	2200.8	48804.3	1926.8	94.9
3.0	126.8	1003.8	111.3	152.7	690.0	823.7	7905.8	690.0	24.4
5.0	44.2	528.4	34.8	51.6	284.3	255.5	1767.5	255.5	18.0
Xanthurenic Acid									
Hours	MID	CER	HIP	HYP	PFC	STR	COR	Median	Plasma
0.0	34.5	44.8	87.2	24.5	<LOQ	3.6	44.7	39.6	9.2
0.5	10.5	12.6	13.8	<LOQ	<LOQ	<LOQ	14.1	13.2	81.9
1.0	28.6	16.7	55.0	4.6	31.0	<LOQ	32.1	29.8	34.3
2.0	9.8	8.1	13.4	<LOQ	<LOQ	<LOQ	11.5	10.7	11.8
3.0	24.5	32.0	35.0	30.1	10.6	<LOQ	16.8	27.3	7.3
5.0	17.4	12.6	26.4	<LOQ	<LOQ	<LOQ	12.4	15.0	4.3
3-Hydroxyanthranilic Acid									
Hours	MID	CER	HIP	HYP	PFC	STR	COR	Median	Plasma
0.0	4.2	4.5	<LOQ	35.5	68.5	69.9	<LOQ	35.5	2.9
0.5	6.3	9.3	<LOQ	40.7	59.7	164.3	1.9	25.0	166.9
1.0	7.7	5.2	<LOQ	18.5	56.5	80.9	4.3	13.1	61.7
2.0	3.6	4.2	<LOQ	16.0	38.2	130.2	<LOQ	16.0	15.6
3.0	4.4	3.7	<LOQ	47.3	46.5	127.2	<LOQ	46.5	7.8
5.0	4.2	2.4	<LOQ	19.1	33.0	88.5	<LOQ	19.1	12.3
Quinolinic Acid									
Hours	MID	CER	HIP	HYP	PFC	STR	COR	Median	Plasma
0.0	<LOQ	<LOQ	<LOQ	<LOQ	<LOQ	<LOQ	<LOQ	<LOQ	134.8
0.5	<LOQ	<LOQ	<LOQ	<LOQ	<LOQ	<LOQ	<LOQ	<LOQ	607.1
1.0	<LOQ	<LOQ	<LOQ	<LOQ	<LOQ	<LOQ	<LOQ	<LOQ	395.6

2.0	<LOQ	<LOQ	<LOQ	<LOQ	<LOQ	<LOQ	<LOQ	<LOQ	357.0
3.0	<LOQ	<LOQ	<LOQ	<LOQ	<LOQ	<LOQ	<LOQ	<LOQ	187.0
5.0	<LOQ	<LOQ	<LOQ	<LOQ	<LOQ	<LOQ	<LOQ	<LOQ	158.6

Results - Blood Brain Barrier Analysis and Assessment of Neuroprotection

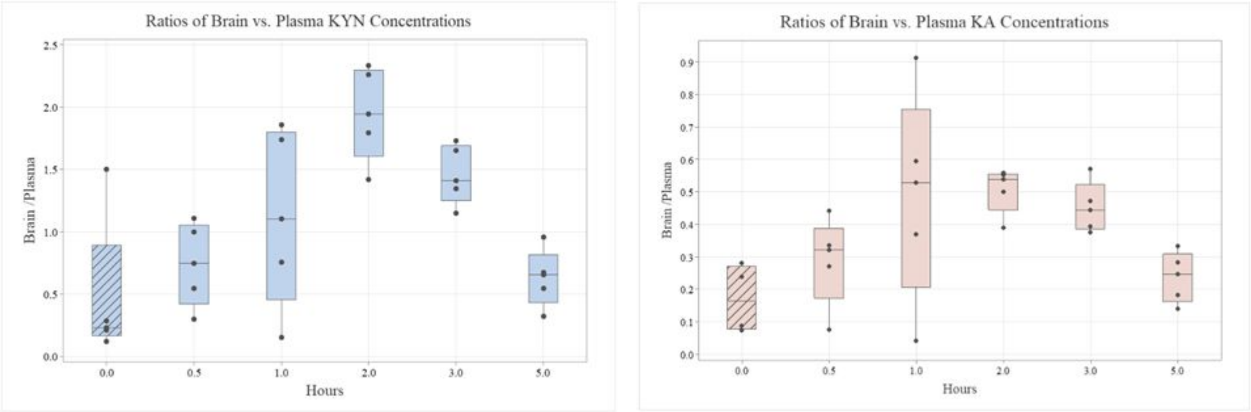

Figure S3. Boxplots visualizing the ratios of brain:plasma KYN concentration (left) and brain:plasma KA concentration (right) measured in rats injected with kynurenine (n = 25), and sacrificed over a period of 5 hours, and rats injected with saline (n = 5) and sacrificed right after injection (0 hours).

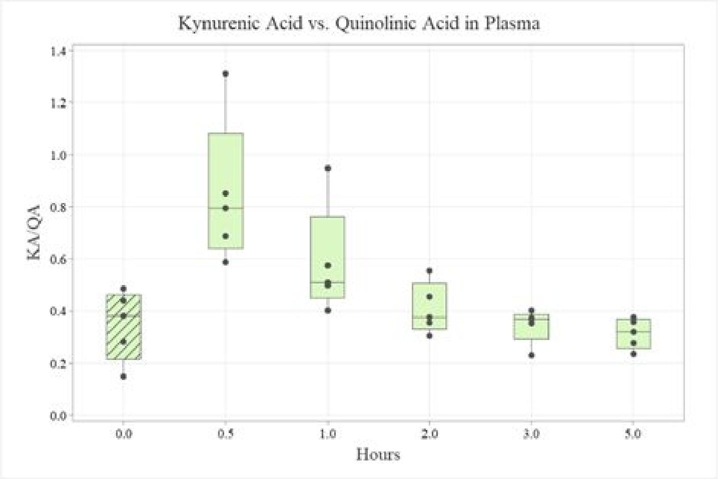

Figure S4. Boxplots visualizing the ratio of kynurenic acid (KA) to quinolinic acid (QA) in plasma. Measured in rats injected with kynurenine (KYN) (n = 25), and sacrificed over a time period of 5 hours, and rats injected with saline (n = 5, shaded boxplot) and sacrificed right after injection (0 hours). KA/QA ratio increased from saline treatment to a median value of 0.8 30 minutes after KYN treatment, indicating that although plasma QA levels are higher than KA levels, KA increases proportionately more than QA following KYN administration

Chronic Study

A

Saline Treatment

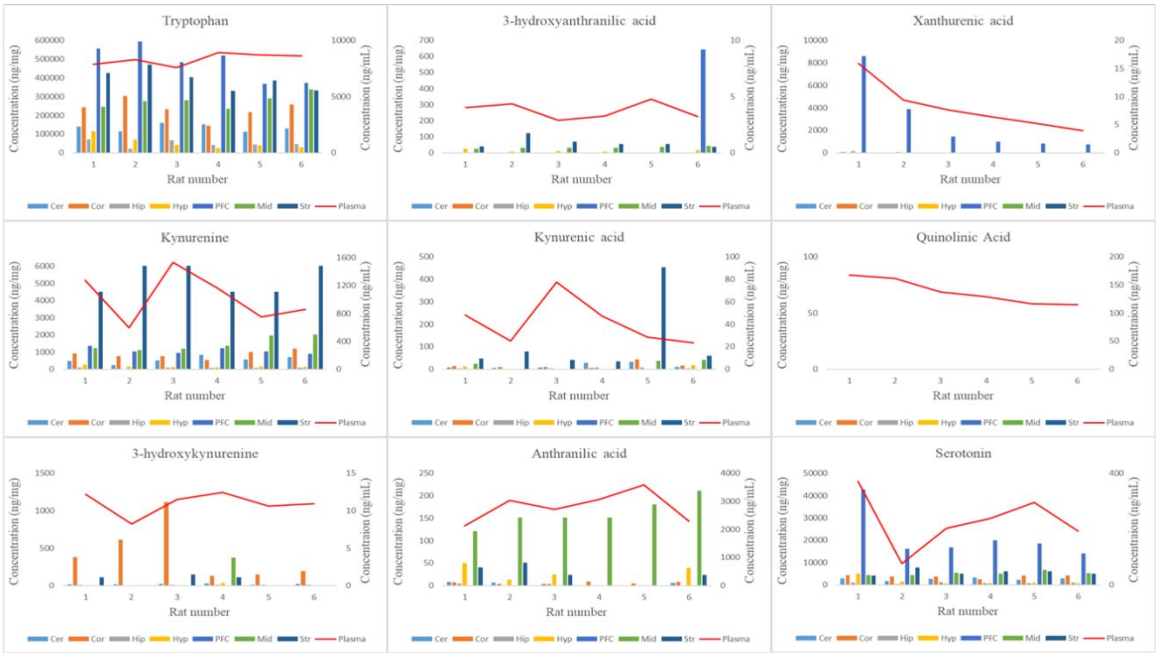

B

Kynurenine Treatment

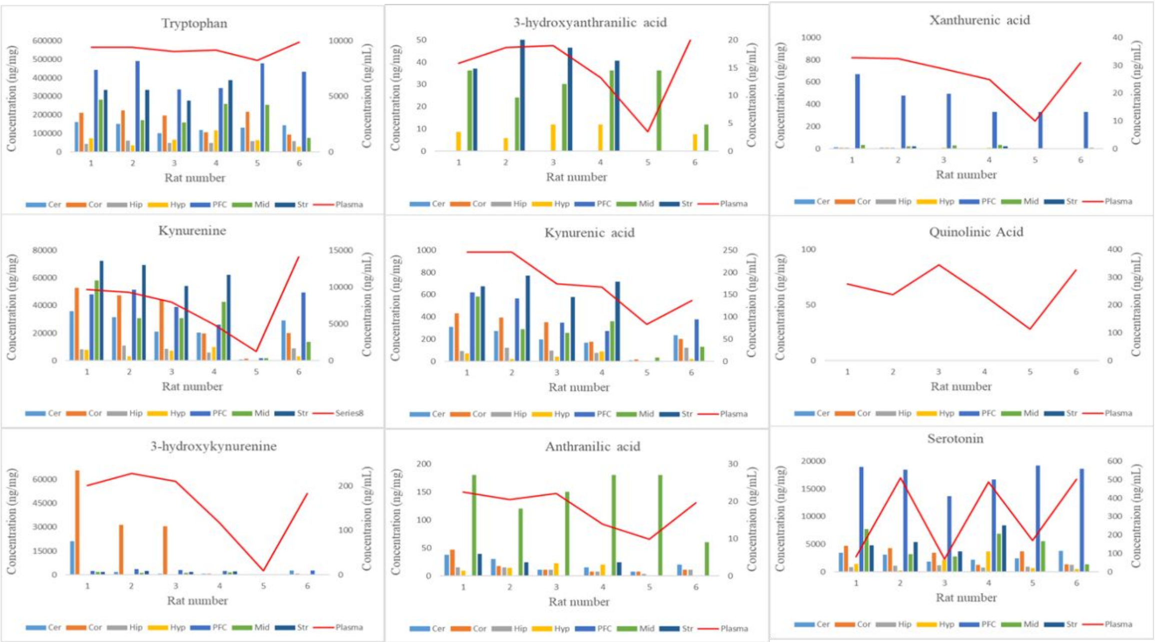

Figure S5. A comparison of concentrations of kynurenine metabolites in various brain regions and plasma of rats treated with saline (Panel A) or treated with kynurenine (Panel B). In Panel A, rats injected with saline served as a control group, showing baseline concentrations of metabolites. Panel B illustrates the changes in metabolite concentrations following a chronic administration of 100 mg/kg kynurenine daily for 14 days, highlighting the effects of prolonged kynurenine treatment.

Table S3. The median concentrations of kynurenine metabolites in different brain regions and plasma between rats administered saline (n=6) and rats administered KYN (n=6). The concentrations in brain regions are expressed as ng/mg, while those in plasma are expressed as ng/ml. Additionally, the fold change was calculated by dividing the average concentration in KYN-treated rats by the average concentration in saline-treated rats. Statistical significance was assessed using the Wilcoxon test (p-values).

		5-HT	TRP	KYN	QA	3-HK	3-HAA	KA	XA	AA
CER	Saline	2832.6	135070.7	537.0	NA	21.0	NA	9.6	15.6	6.6
	Kyn	2783.4	136962.6	25095.0	NA	1358.0	NA	216.4	5.7	17.5
	Fold change	1.1	1.0	41.7	NA	222.1	NA	12.9	0.3	2.8
	p value	0.715	0.955	0.002	NA	0.004	NA	0.015	0.130	0.008
COR	Saline	4012.7	237448.5	841.5	NA	287.0	NA	12.1	16.4	6.0
	Kyn	3618.7	203985.4	32184.1	NA	15692.9	NA	278.4	9.3	11.0
	Fold change	0.8	0.8	35.9	NA	50.1	NA	16.1	0.5	2.7
	p value	0.699	0.041	0.002	NA	0.052	NA	0.004	0.864	0.027
HIP	Saline	862.1	46034.0	95.2	NA	8.0	NA	2.8	20.1	4.3
	Kyn	1055.8	53065.2	8310.5	NA	285.4	NA	96.8	7.4	10.8
	Fold change	1.2	1.1	84.0	NA	33.0	NA	20.8	0.2	2.4
	p value	0.212	0.498	0.002	NA	0.041	NA	0.009	0.020	0.012
HYP	Saline	985.0	40523.1	130.5	NA	37.7	9.7	15.1	15.1	32.2
	Kyn	1085.2	65380.6	5310.4	NA	250.9	8.6	42.5	12.1	11.7
	Fold change	0.9	1.2	35.5	NA	7.0	0.7	3.3	0.8	0.4
	p value	0.774	0.488	0.001	NA	0.005	0.630	0.025	0.923	0.493
MID	Saline	17723.9	501031.9	1024.2	NA	NA	642.1	NA	1228.3	NA
	Kyn	18503.4	437421.1	43488.7	NA	2834.3	NA	376.5	405.5	NA
	Fold change	0.8	0.9	33.3	NA	NA	NA	NA	0.2	NA
	p value	0.937	0.246	0.002	NA	0.004	NA	0.005	0.015	NA
PFC	Saline	5112.3	277361.5	1285.2	NA	377.1	30.2	36.2	30.2	150.8
	Kyn	4395.1	213472.0	30850.4	NA	1450.8	33.2	274.1	30.2	165.9
	Fold change	0.9	0.7	20.1	NA	3.9	0.9	8.1	0.9	0.9
	p value	0.373	0.102	0.003	NA	0.061	0.868	0.010	0.366	0.868
STR	Saline	5546.4	393560.2	5279.7	NA	113.1	55.4	53.9	24.1	32.3
	Kyn	5097.0	335067.0	65731.6	NA	1965.1	43.6	697.3	24.1	24.1
	Fold change	1.0	0.9	12.2	NA	16.4	0.8	5.8	1.0	0.8
	p value	0.166	0.092	0.371	NA	0.157	0.089	0.378		0.446
Pls	Saline	220.3	8443.0	1011.5	133.0	11.2	3.6	38.1	7.0	2875.3
	Kyn	327.7	9278.0	8612.1	256.6	191.5	17.2	171.0	29.8	20.1
	Fold change	1.3	1.1	7.6	1.9	14.4	4.1	4.2	3.3	0.0
	p value	0.872	0.021	0.001	0.009	0.041	0.015	0.0002	0.004	< 0.0001

Results for the Gene Expression Analysis

Acute Study

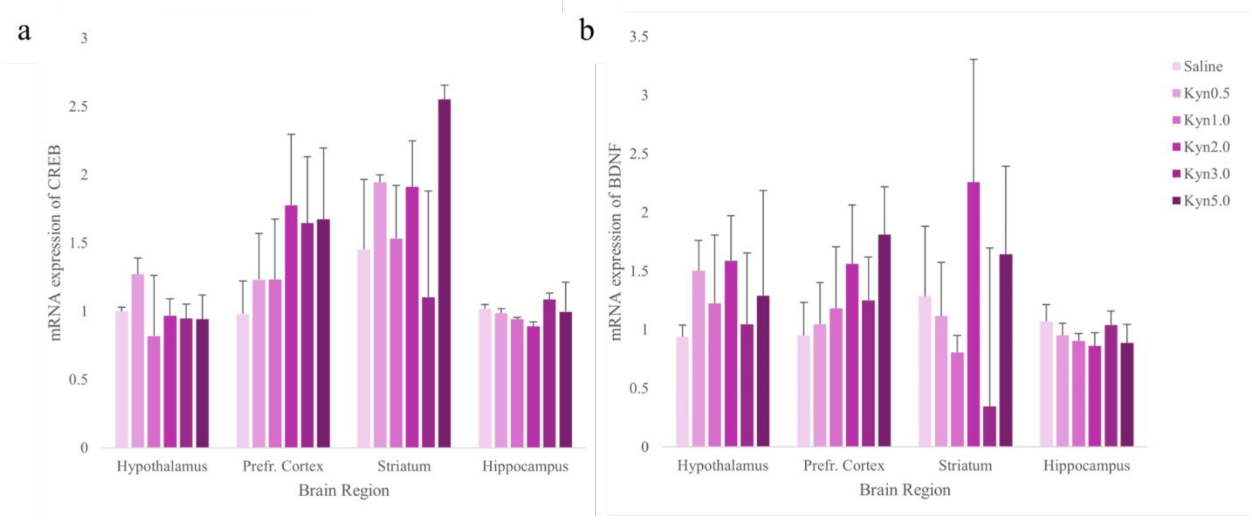

Figure S6. Results of the RT-PCR of (a) CREB and (b) BDNF for the acute study. Results are depicted as columns for each brain region (hypothalamus (HYP), prefrontal cortex (PFC), striatum (STR) and hippocampus (HIP)), and time point of the acute study, medians are reported. Error bars (positive) are given as the standard deviation. 2-Sample t-tests were performed to test for significant differences between saline-treated (Saline) and Kyn-treated (Kyn0.5, Kyn1.0, Kyn2.0, Kyn3.0, Kyn5.0) rats. The tests were performed with the following parameters: normalized data (log transformation, pareto scaling), parametric, equal group variance, unpaired, and FDR threshold of 0.05. Significant differences were considered with a p-value < 0.05. The results showed a significant increase in CREB levels in the hypothalamus from saline to Kyn0.5, and a significant decrease between saline and Kyn2.0 in the hippocampus. BDNF levels significantly increased between saline and Kyn0.5 in the hypothalamus.

Chronic Study

a b

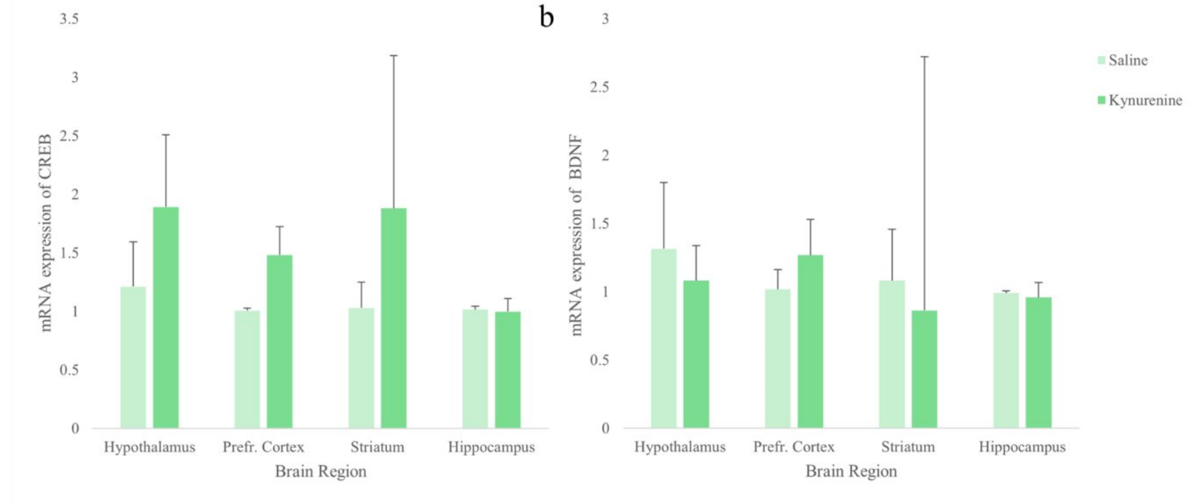

Figure S7. Results of the RT-PCR of (a) CREB and (b) BDNF for the chronic study. Both are depicted as columns for each brain region, showing the results of saline- and kynurenine-treated rats. Error bars (positive) are given as the standard deviation of the brain regions. 2-Sample t-tests were performed to test for significant differences between saline-treated (Saline) and Kyn-treated (Kynurenine) rats. The tests were performed with the following parameters: normalized data (log transformation, pareto scaling), parametric, equal group variance, unpaired, and FDR threshold of 0.05. Significant differences were considered with a p-value < 0.05. CREB and BDNF showed no significant differences in any brain region between saline and kynurenine treatment.
